# Parental Catastrophizing Partially Mediates the Association between Parent-Reported Child Pain Behavior and Parental Protective Responses

**DOI:** 10.1155/2014/751097

**Published:** 2014-01-20

**Authors:** Shelby L. Langer, Joan M. Romano, Lloyd Mancl, Rona L. Levy

**Affiliations:** ^1^School of Social Work, University of Washington, Box 354900, 4101 15th Avenue NE, Seattle, WA 98105, USA; ^2^Psychiatry and Behavioral Sciences, School of Medicine, University of Washington, Box 356560, 1959 Pacific Street, Seattle, WA 98195, USA; ^3^Oral Health Sciences, School of Dentistry, University of Washington, Box 357475, 1959 NE Pacific Street, Seattle, WA 98195, USA

## Abstract

This study sought to model and test the role of parental catastrophizing in relationship to parent-reported child pain behavior and parental protective (solicitous) responses to child pain in a sample of children with Inflammatory Bowel Disease and their parents (*n* = 184 dyads). Parents completed measures designed to assess cognitions about and responses to their child's abdominal pain. They also rated their child's pain behavior. Mediation analyses were performed using regression-based techniques and bootstrapping. Results supported a model treating parent-reported child pain behavior as the predictor, parental catastrophizing as the mediator, and parental protective responses as the outcome. Parent-reported child pain behavior predicted parental protective responses and this association was mediated by parental catastrophizing about child pain: indirect effect (SE) = 2.08 (0.56); 95% CI = 1.09, 3.30. The proportion of the total effect mediated was 68%. Findings suggest that interventions designed to modify maladaptive parental responses to children's pain behaviors should assess, as well as target, parental catastrophizing cognitions about their child's pain.

## 1. Introduction

Catastrophizing cognitions regarding pain have emerged as important predictors of pain-related outcomes [1]. Research spanning both child and adult samples suggests that catastrophizing amplifies pain experience, expressivity, and pain-related dysfunction [2]. For example, catastrophizing among adults has been associated with increased pain intensity, severity and interference, increased pain behavior, decreased pain tolerance, increased depression and distress, and increased disability [3–10]. Similarly, catastrophizing among children has been associated with increased pain intensity, severity and pain-related attentional avoidance, increased pain anxiety and pain behavior, increased depression and distress, increased functional disability, and decreased health-related quality of life [11–20].

From a theoretical standpoint, catastrophizing has been conceptualized as an appraisal process, a cognitive coping strategy, and a means of coping via elicitation of communal support [2]. In a critical review of the literature on catastrophizing, Quartana and colleagues [2] note that pain catastrophizing in particular is characterized by the tendency to magnify the threat value of a pain stimulus, to feel helpless in the face of pain, and by difficulty inhibiting pain-related thoughts. The assessment of catastrophizing has been conducted primarily through self-report questionnaires such as the Coping Strategies Questionnaire [21] and the Pain Catastrophizing Scale (PCS; [22]). Factors within the larger category of catastrophizing have been derived from the PCS, indicating dimensions of rumination, magnification, and helplessness [22]. More recently, attention to catastrophizing among significant others such as spouses/partners or parents of those with pain has led to the development of instruments such as the PCS-Spouse [23] and PCS-Parent forms [24], designed to assess catastrophizing cognitions in response to a loved one's pain.

With these measures, researchers have begun to examine effects that the catastrophizing cognitions of significant others may have on patient outcomes. Relatively recent studies have demonstrated that parents' catastrophic cognitions about their child's pain are associated with adverse outcomes in the child such as increased pain intensity, increased (parent-perceived) pain behavior, increased pain catastrophizing, increased depressive symptomatology, increased disability, and decreased quality of life [15, 16, 19, 25]. Parents' catastrophizing about their child's pain has also been associated with the parents' emotional and behavioral responses to their child's pain. For example, Hechler and colleagues [25] found that both mothers' and fathers' catastrophizing about their child's chronic pain was associated with self-reported solicitous responding to the child's pain (responding in ways that reinforce symptomatology and illness behavior). In a laboratory study in which children were asked to undergo a cold-pressor procedure, parents' catastrophizing about their child's pain was positively associated with their self-reported distress and tendency to want to stop the procedure [26]. In a third study employing a vignette methodology, mothers' catastrophizing about their child's pain was associated with greater endorsement of items reflecting the importance of controlling their child's pain in a hypothetical pain scenario relative to the importance of having their child engage in everyday activities despite the pain [27]. It should be noted, however, that this association only held for hypothetical scenarios involving acute versus chronic pain [27]. Finally, in a naturalistic study of children with leukemia undergoing lumbar puncture and/or bone aspiration procedures, heightened catastrophic thinking about the child's procedural pain was associated with greater distress among parents during the procedure. Increased distress among high catastrophizing parents, moreover, predicted greater pain-attending verbal and nonverbal behavior while interacting with their child after the procedure [28].

**Figure 1:**
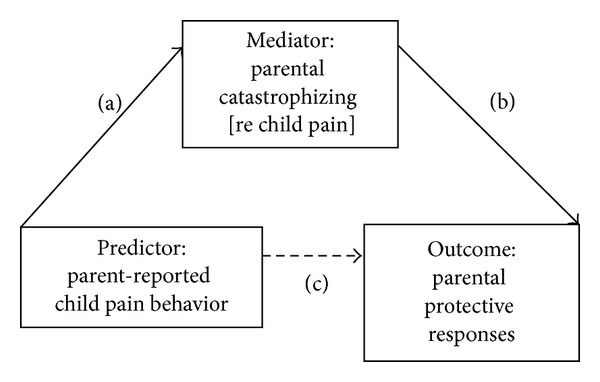
Conceptual model treating parental catastrophizing as a mediator.

While parental catastrophizing appears to be an important factor potentially influencing emotional and behavioral responses to pain in children and their parents, the exact nature of its role remains to be clarified. In a cognitive-behavioral model, cognitive appraisals have been viewed as a mediator between events (or perceived events) and emotional and behavioral responses. Thus, appraisals of threat are seen as activating emotional and behavioral responses to perceived stressors [29]. In this framework, child pain behaviors may serve as triggers that increase the likelihood of catastrophizing cognitions on the part of parents, which in turn may prompt responses to protect the child or mitigate threat. This model is illustrated in Figure 1. We have already outlined evidence for Paths a and b. With respect to Path c, the association between child pain behavior and parental protective responses, results from a heat pain induction experiment indicated that parents engaged in more pain-attending talk with their child if the child talked more about his/her pain [30]. This effect was moderated, moreover, by perceived threat. High catastrophizing parents who were led to believe that the heat induction was threatening engaged in more pain-attending talk (e.g., asking their child, “Does it still hurt?”) than did high catastrophizing parents who were led to believe that the heat induction was neutral (nonthreatening). In contrast, pain-attending talk did not differ as a function of perceived threat among low catastrophizing parents [30].

This model may be particularly relevant in a population such as that of children with Inflammatory Bowel Disease (IBD) and their parents. IBD is the umbrella term for two serious and chronic medical disorders, Crohn's Disease and ulcerative colitis, and is seen with increasing frequency among children [31]. Both conditions are diagnosed after positive medical tests have determined chronic inflammation in the gastrointestinal tract. Clinical features can include malaise, diarrhea, blood and/or mucus in the stool, abdominal pain, anemia, weight loss, fever, abscess, and fistula; malnutrition is common [32, 33]. Extradigestive manifestations may also occur [34]. IBD has been associated with significant personal and societal costs, including depression, anxiety, social deficits, self-management difficulties, poor school functioning, decreased health-related quality of life, and increased health care expenditures [35–40]. The presentation of IBD is frequently variable, marked by symptom flares as well as extended quiescent periods in which few symptoms occur or in which pain behaviors may be infrequent or absent. Increased symptoms, which may or may not indicate underlying increased disease activity, can be accompanied by significant discomfort and pain behavior. Thus, parent perception of pain behaviors may have the effect of triggering or activating threat appraisals and catastrophic cognitions on the part of parents, which could then lead to increased protective responses. In the case of a disease that often presents with a pattern of episodic increases in symptoms and potentially more severe disease activity, it is plausible that parental catastrophizing may be activated by the perception of increased child pain behavior to a greater extent than in chronic pain conditions where symptoms are more constant or where there is no evidence of underlying disease.

**Figure 2:**
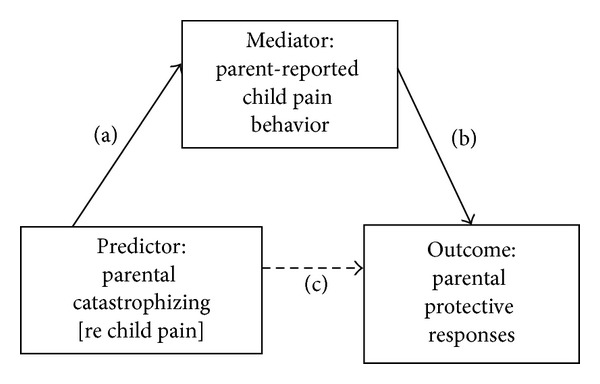
Conceptual model treating parental catastrophizing as a predictor.

An alternate conceptual model is presented in Figure 2. In this model, parental catastrophizing might be viewed as a dispositional, predictive factor leading to parental protective responses, mediated by the perception of pain behavior. Catastrophizing in this context might increase the likelihood that parents perceive pain behaviors on the part of the child, perhaps through heightened sensitivity or vigilance regarding the threat that they represent. In an empirical demonstration of this notion, undergraduates were shown videos of other research participants undergoing a cold-pressor procedure [41]. The undergraduates (termed viewers) were asked to make judgments regarding the level of pain intensity experienced by the participants in the videos. Viewers higher in catastrophizing judged the participants as experiencing more intense pain than did viewers low in catastrophizing. Post-hoc analyses indicated that high catastrophizers were more likely to rely on pain behavior in inferring pain experience; such inferences, however, were not necessarily more accurate. Further evidence comes from a study of school children asked to undergo a pain pressure test [42]. Each child's primary caregiver (mostly mothers) observed this process via a television screen in a separate room. Parents' catastrophizing about their child's pain was associated with higher ratings of their child's pain experience. This effect was independent of their child's observer-coded facial pain expressions, lending support for a “top-down” or observer-driven view of pain perception. Catastrophizing among parents may increase the likelihood of their perceiving child symptoms as a cause for concern or perceiving child behaviors as indicative of pain experience. This may come to influence the responses of parents of children with chronic pain over time.

Very little is known about these processes in pediatric IBD. To our knowledge, only one study has examined parental catastrophic cognitions and protective responses to child gastrointestinal symptoms in the context of IBD. This was a pilot intervention designed to improve coping among female adolescents with IBD and their parents [43]. Treatment consisted of a 1-day workshop followed by several weeks of web-based skill review and practice; sessions targeted, in part, catastrophic cognitions and parental responses, training parents to set appropriate limits and expectations. Families also received communication skills training. Adolescents in the intervention group evidenced prepost treatment reductions in somatic symptoms and improvements in pain coping, and parents reported reductions in catastrophic cognitions and improved (less protective) behavioral responses to their daughter's symptoms. This same group also published a manuscript describing factors associated with attrition from the intervention. Baseline levels of parental catastrophizing were higher among families who dropped out versus completed [44]. While these findings offer important implications for intervention, we still know very little about baseline levels of cognitive and interpersonal processes in this population, not to mention associations among these factors. This study was also small in size (13 assigned to the intervention and 11 assigned to wait-list control) and excluded prepubertal children and male adolescents [43].

In summary, there is only a very small body of literature examining parental catastrophizing and protectiveness and how they relate to child symptoms and functioning in pediatric IBD, a pain-related condition with significant psychosocial costs. In the current study, we tested two models representing parental catastrophizing as either a mediating or predictive factor in relationship to protectiveness, to determine which would be more consistent with data from a sample of children with IBD and their parents.

## 2. Materials and Methods

### 2.1. Participants

One-hundred and eighty-four parent-child dyads served as participants, a subsample of 210 dyads enrolled in a randomized controlled trial (RCT) of a cognitive-behavioral intervention designed to help families manage pediatric IBD. The RCT, still ongoing, is prospective and longitudinal; the present data were collected at baseline, prior to randomization. Of the 210 dyads enrolled, 26 were excluded from our analysis subsample. Twenty families did not complete baseline assessment. One child completed baseline assessment but his/her parent did not. Five additional dyads were randomly excluded to avoid nonindependence among siblings (these five families each had two children with IBD enrolled in the study).

Families were recruited from the gastroenterology departments of Seattle Children's Hospital in Seattle, WA, and Mary Bridge Children's Hospital in Tacoma, WA. All procedures were approved by the Institutional Review Boards of both institutions. Children and parents were included if the child was age 8–17, had a diagnosis of IBD for at least 3 months, was medically approved to engage in normal daily activities, and if the child and parent had cohabited for at least the past 3 months. Exclusion criteria included child chronic disease other than IBD, major surgery in the past year unrelated to IBD, and developmental disability requiring full-time special education or impairing the ability to respond. Participants also needed to be able to speak and comprehend English.

### 2.2. Parent-Reported Measures

Parents completed measures online or, if preferred, via pencil and paper by mail. Questionnaires are described in turn below.

The Pain Catastrophizing Scale-Parent [24] assessed parents' catastrophizing about their child's abdominal pain and other gastrointestinal symptoms. Thirteen items such as “I worry all the time whether my child's pain will end” are rated on a 0–4 scale; summary scores can range from 0–52. The developers reported strong internal consistency (*α* = 0.93) and validity as demonstrated by associations with parent distress and child disability. In our sample, Cronbach's coefficient alpha was 0.92. We used total scores given strong intercorrelations among the subscales and with the total (*r* values ranging from 0.68–0.91, *P* < .001).

The protect subscale of the Adult Responses to Children's Symptoms [45] was used to assess parents' solicitous responses to their children's gastrointestinal symptoms. Exemplar items include “When your child has a stomachache or abdominal pain, how often do you… let him/her stay home from school,” “… bring him/her special treats or little gifts,” and “… get him/her something to eat or drink?” Internal consistency values in the literature range from 0.82 to 0.86 [45–47]. The value based on the present sample was 0.87. Thirteen items are rated on a 0–4 (never to always) scale; summary scores can range from 0–52 [46]. While the measure contains three factor-analytically derived subscales, only the protect subscale has undergone validity testing, associated with a diary version of the subscale, healthcare visits for gastrointestinal symptoms [47], and child pain and functional disability [48].

The Pain Behavior Check List (PBCL; [49]) was used to assess parents' reports of observable expressions of their child's pain. The PBCL was developed for use with adult chronic pain patients and has demonstrated satisfactory reliability and validity [49]; psychometric properties were also established using a nonclinical college sample [50]. We adapted the PBCL for use by parents in assessing child pain behaviors. Most pediatric pain behavior measures were developed in the context of assessing behavior in acute procedural pain situations or acute injury/illness contexts, or to be completed by trained observers [51] and thus were not applicable in the assessment of chronic pain behaviors by parents. In adapting the PBCL for this study, certain items were modified for use with our pediatric/adolescent sample. For example, “take pain medication” was changed to “take medicine,” “walk with a limp or distorted gait” was changed to “walk with a limp or in a different way than usual,” and “use a cane or some other prosthesis” was omitted. We also added “lie down.” All items were rated for frequency on a 0–4 (never to always) scale. The developer reported a Cronbach's coefficient alpha of 0.85 for the total scale; the value based on the present dataset was 0.89. We also used our sample to examine validity. Total PBCL scores were positively correlated with parent-reported child functional disability (*r* = .32, *P* < .001) and child-reported functional disability (*r* = .35, *P* < .001) using the Functional Disability Inventory [52, 53]; child-reported gastrointestinal symptom severity (*r* = .17, *P* = .025) using the Children's Somatization Inventory [54]; and child-reported massaging/guarding the painful area (*r* = .18, *P* = .014), a subscale of the Pain Response Inventory [55]. Conversely, total PBCL scores were inversely correlated with parent-reported child quality of life (*r* = −.42, *P* < .001) and child-reported quality of life (*r* = −.34, *P* < .001) using the Pediatric Quality of Life Inventory [56].

### 2.3. Child-Reported Measures

Children completed questionnaires via phone, administered by a nurse researcher. To facilitate comprehension, answer choices were mailed to children in advance of the phone session. We focus here on the Faces Pain Scale-Revised [57], a validated single-item measure of current pain intensity. Children are shown a row of 6 line-drawn faces. The left-most face depicts no pain, with the faces depicting more and more pain as they move from left to right. Children are instructed to choose the face that shows “how much they hurt right now.” Options are scored as 0 (no pain) to 10 (very much pain). In our sample, this item correlated positively with children's ratings of the extent to which they were bothered by pain in their stomach or abdomen in the past two weeks (*r* = 0.38, *P* < .001), an item contained in the Children's Somatization Inventory [54].

### 2.4. Analyses

Descriptive statistics and correlation analysis were used to describe the sample with respect to demographics and key study variables. Mediation analysis was performed using Hayes' PROCESS macro, a regression-based path analytic technique. Bootstrap methods were used to test for an indirect effect and to compute bias-corrected confidence intervals for this effect [58]. Child age, gender, and current pain were included as covariates in all mediation models. Analyses were conducted using IBM Statistical Package for the Social Sciences 20.0.

## 3. Results

### 3.1. Sample Characteristics

Table 1 presents demographic characteristics of parents and children. Parents were, on average, 44 years old. The majority were mothers, non-Hispanic, and Caucasian. Almost one-half had earned a four-year college degree, 79% were married, and 44% were employed full-time. Their children were, on average, 13.7 years old. Child gender was fairly evenly distributed (47% female and 53% male). Approximately two-thirds had a diagnosis of Crohn's Disease and one-third had a diagnosis of ulcerative colitis.

Table 2 displays descriptive statistics for study variables in addition to a correlation matrix. With respect to bivariate associations, gender was associated with child pain behavior such that girls were judged by their parents to exhibit more pain behavior than were boys. Child age and pain intensity were unrelated to key study variables. Parent-reported child pain behavior, parent catastrophizing, and parent protective responses were all significantly positively associated with each other.

### 3.2. Mediation Analyses

Table 3 displays results of the mediation analyses treating parent-reported child pain behavior as the predictor, parental catastrophizing as the mediator, and parental protective responses as the outcome (Figure 1). Parent-reported child pain behavior was significantly related to both parental protective responses (Path c, estimate = 3.04, *P* < .01) and parental catastrophizing (Path a, estimate = 7.07, *P* < .001). Parental catastrophizing was significantly related to parental protective responses (Path b, estimate = 0.32, *P* < .001). The mediation hypothesis was supported by a significant indirect effect as indicated by the confidence interval excluding zero (Path a × b, estimate = 2.08; 95% CI with 10,000 resamples = 1.09, 3.30). When both the predictor and mediator were entered into the model, Path b remained significant, whereas the effect of parent-reported child pain behavior on protective responses was no longer significant (Path c′, estimate = 0.96, *P* > .05). The ratio of the indirect effect to the total effect or the proportion of the total effect mediated was 68%, indicating support for partial mediation.

Table 4 displays results of mediation analyses treating parental catastrophizing as the predictor, parent-reported child pain behavior as the mediator, and protective responses as the outcome. Parental catastrophizing was significantly related to both parental protective responses (Path c, estimate = 0.32, *P* < .001) and parent-reported child pain behavior (Path a, estimate = 0.03, *P* < .001). Parent-reported child pain behavior was significantly related to parental protective responses (Path b, estimate = 3.04, *P* < .01). However, the hypothesis of parent-reported child pain behavior as a mediator was not supported as indicated by a nonsignificant indirect effect (Path a × b, estimate = 0.02; 95% CI with 10,000 resamples = −0.02, 0.07). When both the predictor and mediator were entered into the model, the effect of parental catastrophizing on protective responses remained significant (Path c′, estimate = 0.29, *P* < .001).

## 4. Discussion

This study sought to test two conceptualizations of the role of parental catastrophizing in relation to parent-reported child pain behavior and parental responses to children's stomachaches and other gastrointestinal symptoms in children with IBD. We found that parent-reported child pain behavior predicted protective responses and this association was partially mediated by parental catastrophizing. An alternative mediation model treating parental catastrophizing as the predictor, parent-reported child pain behavior as the mediator, and protective responses as the outcome was not supported. At least for the present IBD sample, then, the data supported a model in which catastrophic cognitions may be activated in response to perceived child pain behaviors, and such cognitions, in turn, are associated with protective responses.

Findings highlight the importance of parents' cognitions in influencing responses to their children's pain behaviors and suggest directions for the design of psychosocial interventions targeted toward parents as a possible means of influencing child pain behavior and functioning. Our research group has previously demonstrated efficacy of a cognitive-behavioral intervention for children with functional (i.e., recurrent, unexplained) abdominal pain and their parents [59, 60]. An important goal of the cognitive component was reduction of pain-related catastrophic cognitions among both children and parents. A social learning component encouraged parents to respond to their children's symptoms in ways that encouraged activity and reinforced wellness versus illness behavior, important given research demonstrating links between parental solicitous responses to child gastrointestinal symptoms and child symptom severity and disability [13, 61, 62]. The present findings support this approach.

The present findings also offer specific implications for research-based interventions in the context of pediatric IBD. One of these is that reduction of parental catastrophizing in response to child pain behaviors in this population should be considered an important treatment goal. Parents could be trained in fairly standard cognitive methods, such as “look at the evidence for…” or “try practicing alternative thoughts” to manage catastrophizing. It might also be helpful to have parents examine cognitions that arise in response to specific situational cues and to role-play responses to these cognitions, such as reviewing guidelines for when it is appropriate to call the child's physician versus engage in self-management. In addition to targeting and reframing catastrophic cognitions directly, interventions could target the impact or consequences of catastrophizing. For parents, this might include managing their own stress or distress in ways that enable them to respond more adaptively to their child [63].

Given the importance of parental cognitions and behaviors, another implication of the present findings is that interventions targeted only to parents, as opposed to parent-child dyads, might be effective in improving outcomes in children with pain and other symptoms where environmental factors can play a key role in pain expression and ability to cope with symptoms. Such an intervention might focus on decreasing catastrophizing in parents as well as teaching parents strategies to manage their own distress or to minimize maladaptive responses to child distress and pain behaviors. This approach might provide an avenue for simpler, cost-effective, and more efficient methods of treatment delivery.

As stated previously, this study utilized baseline data from a cognitive-behavioral intervention study for children with IBD and their families. As this trial is still underway and we do not yet know whether the intervention produced improved outcomes such as reduced child gastrointestinal symptom severity, we cannot yet test mechanisms or mediators of outcome such as whether changes in parent or child cognitions, or parent responses mediate treatment effects if found. Because IBD has a well-established etiology with significant morbidity, parental appraisals regarding child pain and prognosis occur in a different context than appraisals of parents of children with abdominal pain with no known physiological etiology (functional abdominal pain). However, it is also quite possible that interpersonal factors affecting a child's ability to cope with illnesses with and without well-established etiologies may be similar.

Several limitations of the present study should be addressed. First, we relied on parent-report measures of child pain behavior and parental response. Future research will benefit greatly from the inclusion of both child-report indicators of parent behavior and *objective* measures of both child and parent behaviors, assessed in a naturalistic or in vivo pain situation. Relatedly, our measure of pain behavior (the PBCL) was developed using an adult sample. While internal consistency for our sample was good and the scale correlated positively with measures of child disability and pain response, formal psychometric evaluation of a parent/child adaptation of this measure has not been conducted. Use of a child-specific, and perhaps even abdominal pain-specific, measure of pain behavior is advised. Second, our sample was comprised largely of mothers and results may not generalize to fathers. Mothers report higher levels of catastrophizing than fathers [25]. Replication in a more evenly distributed sample is advised. Third, given the cross-sectional and associational nature of our data, we cannot infer directionality, sequence, or causality. Parent-reported child pain behavior and parental protective responses were measured concurrently and were positively correlated. Thus, it is not surprising that when we tested a reverse mediation model treating protective responses as the predictor, parental catastrophizing as the mediator, and parent-reported child pain behavior as the outcome, our data supported this model, lending further support for a linkage between these two outcomes through parental catastrophizing when factors are measured contemporaneously. Future research is needed, however, to clearly elucidate directionality of Path c. Investigations utilizing a repeated measures design are warranted to explicate timing and sequence, as done in observational studies of patients and spouses in the adult chronic pain arena [64].

## 5. Conclusions

Despite these limitations, this study provides evidence for the importance of parental catastrophizing cognitions as potential mediators of the relationship between perceived child pain behavior and parental protective responses. Should such findings be replicated in further studies using longitudinal designs, they would strengthen the rationale for targeting reduction of parental catastrophizing in cognitive-behavioral treatments aimed at improving family coping and child functioning for children with chronic gastrointestinal illness.

## Figures and Tables

**Table 1:** Sample characteristics (*n* = 184 dyads).

Characteristic	Parent	Child
Age, M (SD)	44.37 (6.85)	13.72 (2.72)
Age, range	27–67	8–18
Gender, *n* (%) female	166 (90.2)	87 (47.3)
Ethnicity, *n* (%) Hispanic	3 (1.6)	8 (4.3)
Race, *n* (%) Caucasian	171 (92.9)	162 (88.0)
Education, *n* (%) 4-year college degree or higher	90 (48.9)	—
Employment status, *n* (%) employed full-time	81 (44.0)	—
Marital status, *n* (%) married	145 (78.8)	—
Disease, *n* (%)		
Crohn's Disease	—	126 (68.5)
Ulcerative colitis	—	58 (31.5)
Time since diagnosis in years, M (SD)	—	2.30 (2.41)

**Table 2:** Correlations among study variables and descriptive statistics (*n* = 184).

	1	2	3	4	5	6	M (SD)	Scale
(1) Child age	1.00	.02	.02	−.11	-.01	−.08	13.72 (2.72)	NA
(2) Child gender (M 1, F 2)		1.00	−.02	.18*	.00	−.01	NA	NA
(3) Child current pain			1.00	.04	.02	.04	0.51 (1.46)	0–10
(4) Parent-reported child pain behavior				1.00	.42**	.24**	1.53 (0.66)	0–4
(5) Parental catastrophizing					1.00	.40**	20.55 (10.77)	0–52
(6) Parental protective responses						1.00	22.41 (8.65)	0–52

Note: *P < .05 and **P < .01.

**Table 3:** Results of mediation analyses treating parent-reported child pain behavior (PRCPB) as the predictor, parental catastrophizing (CAT) as the mediator, and protective responses as the outcome (*n* = 184).

Path	Effect	Estimate (SE)	*P*	95% CI
c	Effect of PRCPB on protect	3.04 (0.97)	0.002	—
a	Effect of PRCPB on parent CAT	7.07 (1.12)	<0.001	—
b	Effect of parent CAT on protect	0.32 (0.05)	<0.001	—
a × b	Indirect effect	2.08 (0.56)	—	1.09, 3.30
c′	Direct effect	0.96 (1.01)	0.342	

Note: confidence intervals excluding zero indicate statistical significance. The ratio of the indirect effect (a × b) to the total effect (c) or the proportion of the total effect mediated was 0.68.

**Table 4:** Results of mediation analyses treating parental catastrophizing (CAT) as the predictor, parent-reported child pain behavior (PRCPB) as the mediator, and protective responses as the outcome (*n* = 184).

Path	Effect	Estimate (SE)	*P*	95% CI
c	Effect of CAT on protect	0.32 (0.05)	<0.001	—
a	Effect of CAT on PRCPB	0.03 (0.004)	<0.001	—
b	Effect of PRCPB on protect	3.04 (0.97)	0.002	—
a × b	Indirect effect	0.02 (0.03)	—	–0.03, 0.07
c′	Direct effect	0.29 (0.06)	<0.001	—

Note: confidence intervals excluding zero indicate statistical significance. The proportion of the indirect effect (a × b) to the total effect (c) or the proportion of the total effect mediated was 0.08 (0.0245/0.3184).
